# Influence of scene aspect ratio and depth cues on verticality perception bias

**DOI:** 10.1167/jov.24.7.12

**Published:** 2024-07-19

**Authors:** Kanon Fujimoto, Hiroshi Ashida

**Affiliations:** 1 Department of Psychology, Graduate School of Letters, Kyoto University, Japan

**Keywords:** horizon, scene, spatial orientation, subjective visual vertical, virtual reality

## Abstract

Perceiving verticality is crucial for accurate spatial orientation. Previous research has revealed that tilted scenes can bias verticality perception. Verticality perception bias can be represented as the sum of multiple periodic functions that play a role in the perception of visual orientation, where the specific factors affecting each periodicity remain uncertain. This study investigated the influence of the width and depth of an indoor scene on each periodic component of the bias. The participants were presented with an indoor scene showing a rectangular checkerboard room (Experiment 1), a rectangular aperture on the wall (Experiment 2), or a rectangular dotted room (Experiment 3), with various aspect ratios. The stimuli were presented with roll orientations ranging from 90° clockwise to 90° counterclockwise. The participants were asked to report their subjective visual vertical (SVV) perceptions. The contributions of 45°, 90°, and 180° periodicities to the SVV error were assessed by the weighted vector sum model. In Experiment 1, the periodic components of the SVV error increased with the aspect ratio. In Experiments 2 and 3, only the 90° component increased with the aspect ratio. These findings suggest that extended transverse surfaces may modulate the periodic components of verticality perception.

## Introduction

The perception of verticality is crucial for accurate spatial orientation, especially for bipeds such as humans. In addition to vestibular and proprioceptive stimuli, the visual environment is rich in vertical cues such as the orientation and spatial layout of daily objects. Our visual reliance on verticality perception is demonstrated by the rod-and-frame illusion (Witkin & Asch, 1948), in which a vertical line segment (rod) appears to tilt in the opposite direction of the surrounding tilted frame. This illusion is believed to be caused by a shift in the subjective vertical relative to the frame orientation (Dassonville & Reed, 2015; Ebenholtz & Benzschawel, 1977; Sigman, Goodenough, & Flannagan, 1979). The extent of the rod-and-frame illusion is measured by having a participant rotate the rod to appear vertical. The orientation of the adjusted rod relative to the true vertical is referred to as the subjective visual vertical (SVV) error.

In the typical rod-and-frame illusion with a seated participant observing a square frame, the frame orientation modulates the apparent tilt of the rod in a sinusoidal manner with 90° periodicity, no bias at upright, and 45° frame orientations (Alberts, de Brouwer, Selen, & Medendorp, 2016; Alberts, Selen, Verhagen, Pennings, & Medendorp, 2018; Beh, Wenderoth, & Purcell, 1971; Oltman, 1968; Spinelli, Antonucci, Goodenough, Pizzamiglio, & Zoccolotti, 1991). The pattern of the SVV error in a real-life scene is more complicated than for a simple frame (Howard & Childerson, 1994). When the SVV error is plotted as a function of scene orientation, the curve of the SVV error is represented by the sum of multiple periodicities (e.g., 90°, 180°, and 360°) (Fujimoto & Ashida, 2022; Haji-Khamneh & Harris, 2009; Harris, Dyde, & Jenkin, 2007; Harris, Jenkin, Dyde, & Jenkin, 2011; Mittelstaedt, 1986). Each component of periodicity is associated with a type of visual orientation cue (Haji-Khamneh & Harris, 2009; Harris et al., 2007; Harris et al., 2011). For example, frame cues (i.e., floor, ceiling, and walls) could be related to the 90° component of the SVV error, with four potential upward directions. The horizontal cue, which is defined by the line that specifies the elevation of the horizon line, is related to the 180° component, with two potential upright directions. Everyday objects and their spatial relationships provide unique directional cues in the upright direction and could contribute to the 360° component of the SVV error. However, whether or not each visual cue actually influences the corresponding component of periodicities remains unconfirmed.

The visual factors responsible for the 180° component of the SVV error, associated with the horizontal cue, are the least understood because the component can be produced in situations with no explicit horizontal line visible, such as in indoor scenes (Fujimoto & Ashida, 2022; Haji-Khamneh & Harris, 2009). A possible source of the 180° component of an indoor scene is the aspect ratio (e.g., the width and height of the room) because an increase in such aspect ratios produces a rotational periodicity of 180°. However, of the previous studies of the 180° component in scenes lacking a horizon line, neither Fujimoto and Ashida (2022) nor Haji-Khamneh and Harris (2009) controlled the aspect ratio of the room, and Fujimoto and Ashida (2022) presented a rectangular room with a 2:1 aspect ratio. Moreover, depth information may also be important for the 180° component. Previous research that presented a line segment as an illusion inducer reported only the 90° component, although the inducer itself had 180° rotational periodicity (Li & Matin, 2005a; Vingerhoets, De Vrijer, Van Gisbergen, & Medendorp, 2009). These studies suggest that depth information is important for inducing the 180° component, but whether depth information has this effect on each visual component has yet to be examined.

Thus, the present study investigated the contribution of width and depth information (transverse plane) in an indoor scene to the periodic components of the SVV error using three spatial structures. In Experiment 1, using a head-mounted display (HMD), we presented a scene of an unfurnished room with checkerboard patterns on interior surfaces with various width-to-height aspect ratios. In Experiment 2, we presented a rectangular aperture with various width-to-height aspect ratios on a frontoparallel checkerboard plane to determine whether the change in two-dimensional (2D) components of the scene was sufficient to influence the 180° component. In Experiment 3, we replaced the checkerboard pattern used in Experiments 1 and 2 with a random dot texture to examine whether the line components (linear perspective) affected the 180° component. In each of these three experiments, the participants completed a rod adjustment task to measure SVV error. We performed a frequency analysis for the SVV error to validate the periodic components in the model. We then applied a vector sum model (Dyde, Jenkin, & Harris, 2006; Mittelstaedt, 1983; Mittelstaedt, 1986) to the SVV errors and estimated the visual weights of the periodic components.

## General methods

### Participants

Each participant took part in one of three experiments. Each experiment included 30 participants: Experiment 1, 19 males and 11 females (mean age = 21.20 years, *SD* = 1.51); Experiment 2, 16 males and 14 females (mean age = 21.63 years, *SD* = 2.09); Experiment 3, 18 males and 12 females (mean age = 21.50 years, *SD* = 1.93). The participants were undergraduate or graduate students at Kyoto University with normal or corrected-to-normal visual acuity and no history of vestibular disorders. They were informed of the objective of the study and provided written consent for participation and publication. The study was conducted in accordance with the tenets of the Declaration of Helsinki. The local ethics committee of Kyoto University approved the experimental procedures (2-P-3).

### Apparatus and stimuli

**Figure 1.**
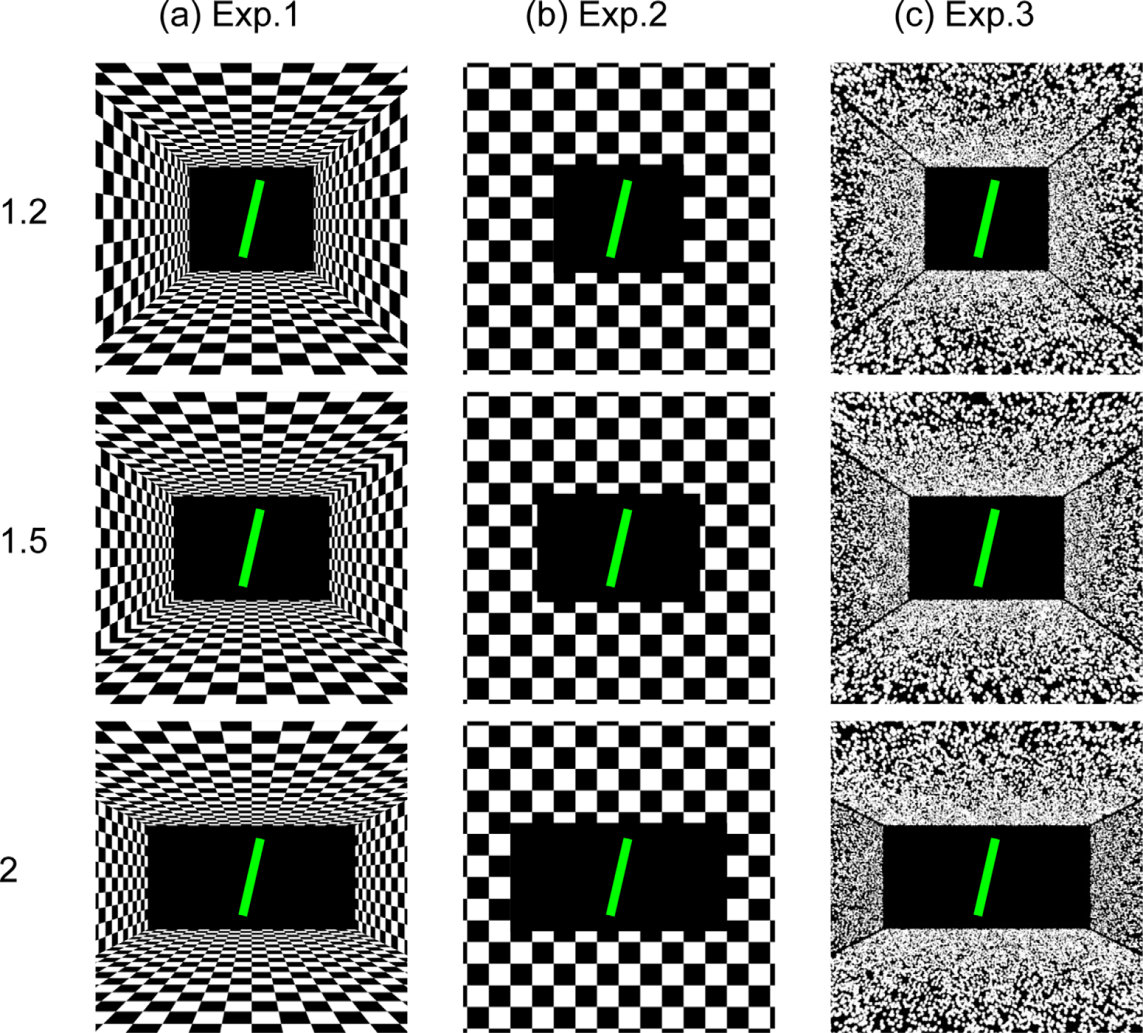
The visual stimuli presented to participants in Experiments 1 to 3. (**a**) In Experiment 1, a virtual room was presented with the three width-to-height aspect ratios of 1.2:1, 1.5:1, and 2:1. (**b**) Experiment 2 showed a frontoparallel wall with different central rectangular apertures. The apertures featured the same three width-to-height aspect ratios as those in Experiment 1. (**c**) In Experiment 3, a random dot texture room was presented with the same aspect ratios as in Experiments 1 and 2. The rooms are presented in upright (0°) positions in the figure. In each image, a green rod was visible 15 meters away from the viewpoint. The rod is enlarged in the figure for clarity.

Visual stimuli were presented on an Oculus Rift CV1 HMD (Meta Technologies, Menlo Park, CA) with a diagonal field of view of approximately 110°. The HMD featured a pair of organic light-emitting diode displays with a resolution of 1080 × 1200 pixels per eye and a refresh rate of 90 Hz. The interpupillary distance between the displays was fixed at 63 mm. To reduce experiment time and physical contact with participants due to COVID-19 restrictions, the interpupillary distance was not adjusted for each individual. Before the experiment, each participant checked the visibility of the displays and adjusted the HMD position until the displays were clearly visible. The experiment was controlled using Windows 10 (Microsoft Corporation, Redmond, WA) on a desktop PC, and the participants’ responses were recorded using an Xbox One gamepad controller (Microsoft Corporation). Visual stimuli were created and controlled using Unity 5.3.1 in three dimensions (Unity Technologies, San Francisco, CA). We presented three virtual scene types (Figure 1). In Experiment 1, the visual stimulus was a simulation of a room with a checkerboard interior pattern, with a height of 8 meters and a depth of 20 meters (Figure 1a). This was presented binocularly. The room featured three width-to-height aspect ratios: 1.2:1 (8 × 9.6 meters; 21.3° × 25.6°), 1.5:1 (8 × 12 meters; 21.3° × 32°), and 2:1 (8 × 16 meters; 21.3° × 42.6°). A virtual camera that simulated the binocular viewpoint of the participant was placed at the center of the side walls, 20 meters away from a black back wall. The size and density of the checkerboard pattern were constant across the aspect ratio conditions, with the sides of each square measuring 0.67 meter (2.23 squares/m^2^). In Experiment 2, we presented a frontoparallel wall at a distance of 20 m from the participant's viewpoint, which covered the entire field of view (Figure 1b). The wall was textured with a checkerboard pattern, with the square size of the surrounding walls (1.6 meters) the same as the mean of retinal size of the square in Experiment 1. In the center of the facing wall was a black rectangular aperture with the same size and aspect ratio as the back wall in Experiment 1. The wall was textured with a random offset. In Experiment 3, we presented a rectangular room comprised of small randomly positioned spheres (Figure 1c). The spheres were white and had no shade. The simulated size of each sphere was 0.1 meter. The room dimensions and three width-to-height aspect ratios were the same as those used in Experiment 1. The background color of the simulated environment was black. The mean luminance of the room was the same as that in Experiment 1.

In each trial of the three experiments, the viewpoint was tilted along the line of sight at one of 19 scene roll orientations ranging from –80° to 90° in 10° increments, to produce a range of different scene orientations. Within the scenes, a green rod, which extended 17° lengthwise and 0.6° widthwise, was presented 15 meters away from the viewpoint.

### Procedure

**Figure 2.**
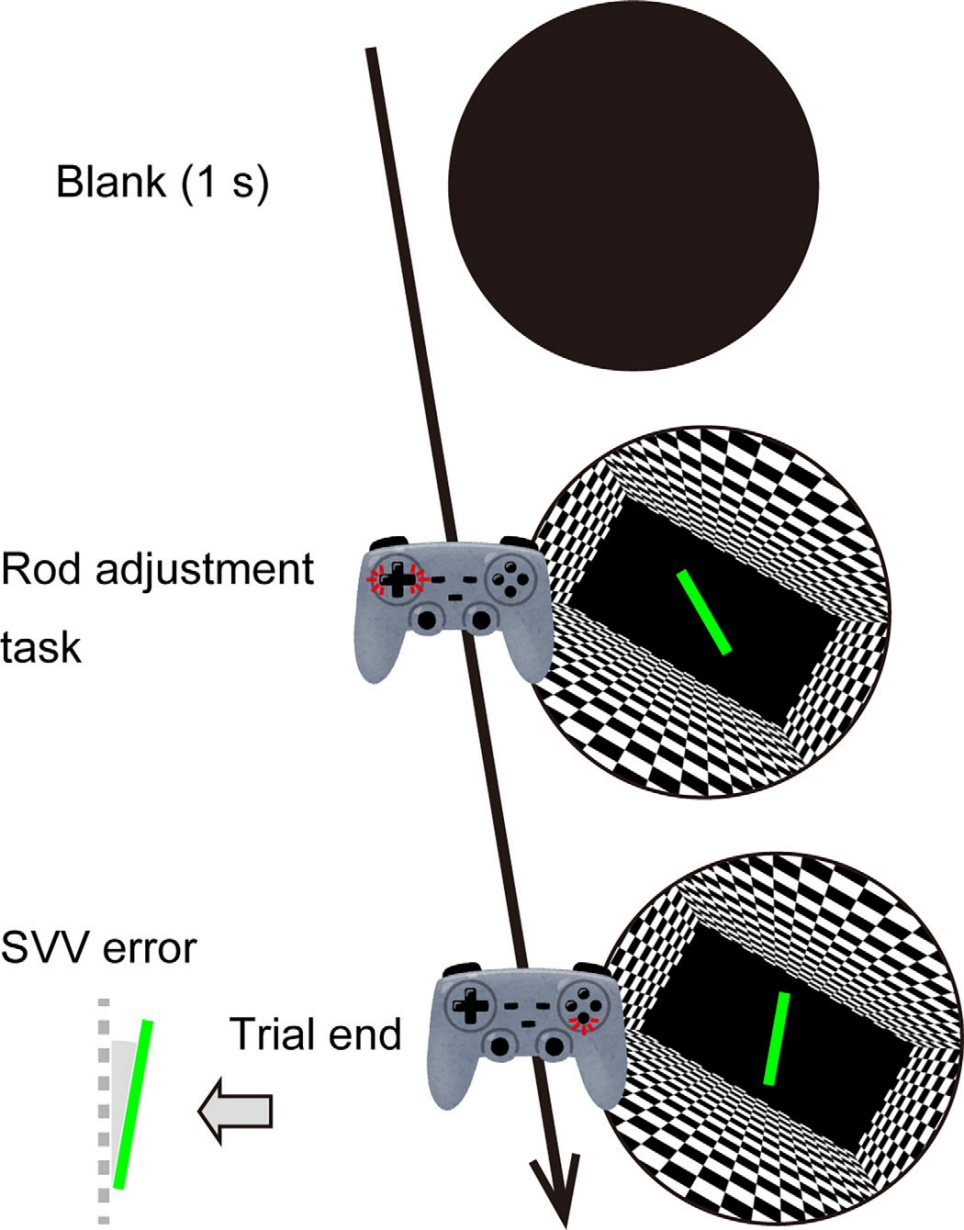
Illustration of the experimental procedure used in each trial. Participants rotated the rod in the image using the left and right gamepad buttons until it appeared gravitationally vertical. When the rod matched the subjective vertical orientation, they pressed the select button. The orientation of the adjusted rod relative to the true vertical was defined as the SVV error. The rod is enlarged in the figure for clarity.

The participants were seated on a stool, and the height was adjusted for each individual. The HMD was worn over the eyes with the participant's head stabilized by a chin rest. The gamepad controller was held in the participant's lap with both hands. For each trial, one of the 19 tilted rooms was presented (Figure 2). The initial rod orientation was randomized in each trial to avoid any influence of initial rod orientation (Hoppenbrouwers, Wuyts, & Van de Heyning, 2004). The participants adjusted the rod orientation such that it appeared gravitationally vertical. When a participant pressed the right or left button on the gamepad, the rod rotated clockwise (CW) or counterclockwise (CCW), respectively, by 2°/s. If the button was held down for more than 200 ms, the rod rotation speed increased to 30°/s. When the rod matched the subjective vertical, the participant pressed the select button. This recorded their response and started the next trial.

The experiment consisted of three blocks of 72 trials. Each block included one of the three room aspect ratios, and the 19 scene roll orientations were each repeated four times. Scene roll orientations were presented in a randomized order, and the order of the room aspect ratios was counterbalanced across participants. After each block, the participant took a break and removed the HMD if needed. Before the experiment, participants practiced several trials to become familiar with the task.

### Data processing, modeling, and analysis

The final adjustments of the rod angles were recorded to produce SVV errors relative to the gravitational vertical (0°) for each trial. The SVV errors were then sorted as a function of scene orientation for each aspect ratio for each participant in each experiment.

Before model analyses, we conducted a frequency analysis to validate the model components. A fast Fourier transform was applied to each participant's SVV error profile for each aspect ratio, and the mean amplitude spectrum across participants was computed.

To assess the effects of visual cues on the SVV error, we used the weighted vector sum model proposed by Mittelstaedt 1983, Mittelstaedt 1986, and Dyde et al. (2006). This represents the orientations of gravity, body, and visual cues as vectors, the respective lengths of which were indicative of their relative weights. The SVV error was assumed to be the sum of these vectors, as follows:

(1) $$\begin{array}{c} s=g+b+v\; \end{array}$$

where ***s***, ***g***, ***b***, and ***v*** indicate the spatial vectors of SVV, gravity, body axis, and visual orientation, respectively. The model assumes that the observer adjusts the rod orientation to ***s***. Because this study examined the SVV error on the frontal plane only, we treated the vectors as two dimensional, with only horizontal and vertical components, ignoring the depth component. The lengths of vectors ***g***, ***b***, and ***v*** represent their relative contributions. The participants were seated upright, and the gravity and body vectors were aligned, so we considered these vectors as a single unit:

(2) $$\begin{array}{c} g+b=\left(\begin{array}{c} 0\\ 1 \end{array}\right)\; \end{array}$$

where the first and second components on the right represent the horizontal and vertical components, respectively. We estimated the lengths of the visual vectors relative to the gravity–body vector.

Previous studies indicate that at least three visual components (90°, 180°, and 360°) contribute to spatial orientation (Fujimoto & Ashida, 2022; Haji-Khamneh & Harris, 2009; Mittelstaedt, 1986). We omitted the 360° component because the visual stimuli had a rotational symmetry of order 2, and the maximum periodicity was 180°. The 45° component was included on the basis of the frequency analysis (see Frequency analysis in Results). Hence, we modeled the effects of the 45°, 90°, and 180° visual vectors, which meant that ***v*** was broken down into three visual components:

(3) $$\begin{array}{c} \begin{array}{c} \;v=v_{45}+v_{90}+v_{180}\\ =w_{45}\left(\begin{array}{c} \cos8\theta \\ \sin8\theta \end{array}\right)+w_{90}\left(\begin{array}{c} \cos4\theta \\ \sin4\theta \end{array}\right)+w_{180}\left(\begin{array}{c} \cos2\theta \\ \sin2\theta \end{array}\right) \end{array}\; \end{array}$$

where ***v*_45_**, ***v*_90_**, and ***v*_180_** represent vectors with 45°, 90°, and 180° periodicities, respectively. Each vector consisted of cosine and sine functions of different periods and weights (lengths) of *w*_45_, *w*_90_, or *w*_180_. θ is the scene orientation in radians.

The equations for these are summarized as follows:

(4) $$\begin{array}{ccc} & & \;s\left(\begin{array}{c} s_{h}\\ s_{v} \end{array}\right)\\ & & \;=\left(\begin{array}{c} w_{45}\cdot \cos8\theta +w_{90}\cdot \cos4\theta +w_{180}\cdot \cos2\theta \\ 1+w_{45}\cdot \sin8\theta +w_{90}\cdot \sin4\theta +w_{180}\cdot \sin2\cdot \theta \end{array}\right)\;\; \end{array}$$

where the two vector elements ***s_h_*** and ***s_v_*** are the horizontal and vertical components of the SVV, respectively. We used the inverse tangent of ***s*** to obtain the angle of orientation:

(5) $$\begin{array}{c} \varphi =\tan^{-1}\frac{S_{h}}{S_{v}}\; \end{array}$$

where φ represents the amount of the SVV error.

We compared three possible models with different component combinations: a full model that included all three vectors, a conventional model that included the 90° and 180° components, and a baseline model with only the 90° component. To compare the maximum likelihood estimates of the models, we used Akaike's information criterion (AIC).

Prior to model fitting, we subtracted the mean of the SVV errors from the data for each room aspect ratio and each participant to omit the overall offset of the data. The original uncentered data are plotted in Supplementary Figure S1. We fitted the three models to the SVV error for each participant and each room aspect ratio using nonlinear least-squares estimates, using the nls function in R 4.0.3 (R Foundation for Statistical Computing, Vienna, Austria).

We obtained up to three parameters of the visual vector (***w***) for each participant and room aspect ratio, which corresponded to the lengths of the three visual vectors. After identifying the best model, we applied a repeated-measures analysis of variance (ANOVA), with periodicity (45°, 90°, and 180°, or portions of them) and aspect ratios (1.2, 1.5, and 2) as within-participant variables. We applied the Greenhouse–Geisser correction to violations of sphericity, where appropriate. Family-wise errors were corrected using Shaffer's modified sequentially rejective Bonferroni procedure. Adjusted *p* values were used for multiple comparisons.

## Results

### Frequency analyses

**Figure 3.**
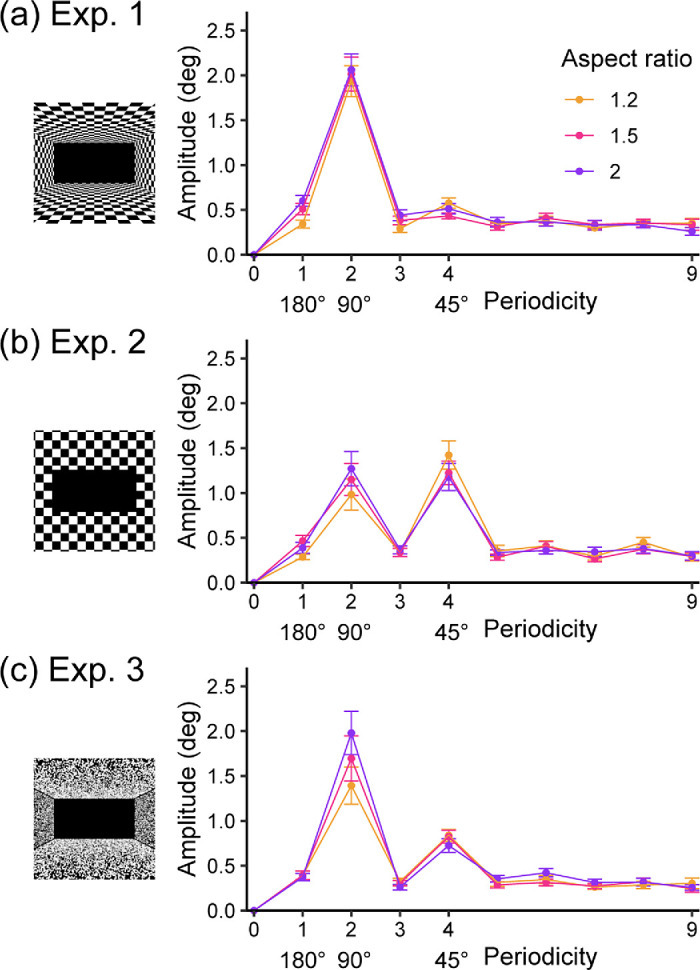
Amplitude spectra of the SVV errors found in the three experiments in this study. The error bars represent the standard error of the mean across participants.

Figure 3 plots the mean amplitude spectrum for each experiment. We found clear peaks at 90° periodicity in all three experiments, which is consistent with previous reports (Alberts et al., 2016; Alberts, 2018; Beh et al., 1971; Oltman, 1968; Spinelli et al., 1991). The 180° periodicity was relatively small, but, in Experiment 1, there was an increase in amplitude as the aspect ratio increased. We also found a peak at 45°, especially in Experiment 2, which was not included in the models used in previous studies.

### Model fitting

Table 1 shows the mean AIC values across participants for each experiment, aspect ratio, and model. The best (lowest) AICs were obtained for the 45° + 90° + 180° model under all conditions. Most of the AIC values for the 90° model were lower than those for the 90° + 180° model. However, the AIC values found in Experiment 1, with larger aspect ratios, were lower in the 90° + 180° model than in the 90° model. We used the 45° + 90° + 180° model to compare the model parameters between experiments.

**Table 1.** Comparison of AIC values found using three different models for each experiment and aspect ratio.

		Mean AIC (*SD*)		
Experiment	Aspect ratio	45° + 90° + 180° model	90° + 180° model	90° model
1	1.2	−301.27 (60.57)	−298.49 (57.57)	−298.98 (57.41)
	1.5	−302.94 (57.59)	−301.67 (55.47)	−300.84 (55.15)
	2	−304.24 (61.2)	−302.3 (60.08)	−299.28 (59.08)
2	1.2	−305 (65.45)	−281.7 (66.08)	−283.31 (65.99)
	1.5	−307.66 (62.37)	−289.23 (62.25)	−289.64 (62.49)
	2	−298.61 (52.08)	−282.54 (51.95)	−283.44 (52.08)
3	1.2	−315.31 (50.29)	−305.71 (46.44)	−306.03 (46.79)
	1.5	−318.36 (44.14)	−308.41 (40.37)	−308.51 (40.56)
	2	−308.17 (53.02)	−301.53 (50.30)	−301.92 (50.10)

### Experiment 1

**Figure 4.**
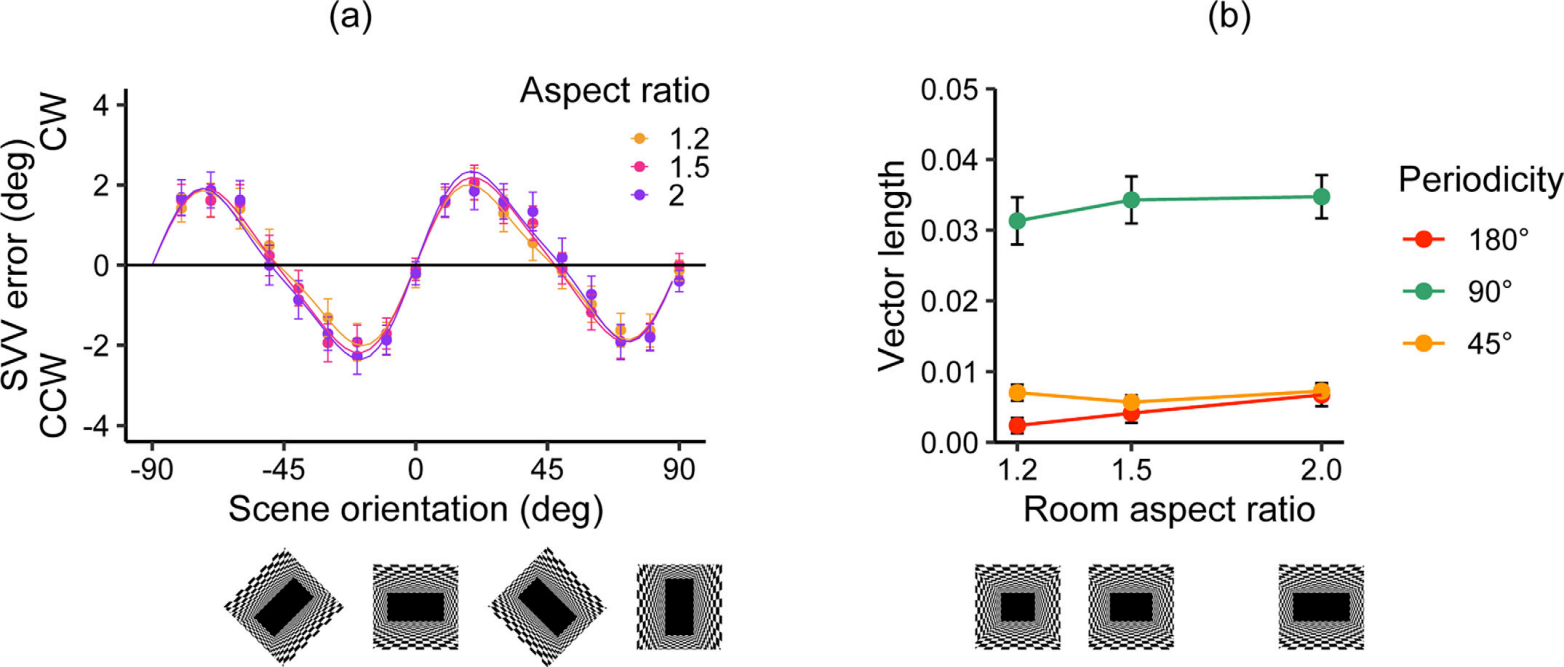
Results of Experiment 1. (**a**) Mean SVV errors as a function of scene orientation. Positive and negative SVV error values correspond to clockwise (CW) or counter-clockwise (CCW) rod adjustments relative to the gravitational vertical, respectively. Data for each aspect ratio were mean centered. The curves demonstrate the best fit of the model to the data. (**b**) Best-fit parameters of the visual vector lengths of the three periodicities for each room aspect ratio. These were obtained for each participant and averaged. Error bars represent the standard errors of the mean across participants.

Figure 4a illustrates the mean SVV errors across participants as a function of scene orientation for each room aspect ratio, where each curved line represents the mean of the best model fits obtained. The mean SVV errors varied as a function of scene orientation and aspect ratio. Each curved line in Figure 4a represents the mean of the best model fit obtained. We also plotted the mean length of each visual vector obtained for each participant and each room aspect ratio (Figure 4b). There was a linear increase in the vector length of the 180° component, whereas the length of the 90° component increased more gently.

A two-way repeated-measures ANOVA with periodicity (45°, 90°, and 180°) and room aspect ratios (1.2, 1.5, and 2) as within-participant variables revealed a significant main effect of room aspect ratio, *F*(1.7, 49.31) = 4.88, *p* = 0.016, η^2^ = 0.004, and periodicity, *F*(1.32, 38.18) = 94.16, *p* < 0.001, η^2^ = 0.566. The interaction between them was not significant, *F*(3.32, 96.19) = 1.46, *p* = 0.227, η^2^ = 0.003. The multiple-comparison tests illustrated that the vector length for the smallest room aspect ratio (1.2) was significantly lower than that for the widest aspect ratio condition, *t*(29) = 2.61, *p* = 0.042, *d* = 0.33. The comparisons between 1.2 and 1.5, *t*(29) = 1.51, *p* = 0.141, *d* = 0.140, and the comparisons between 1.5 and 2, *t*(29) = 1.97, *p* = 0.0582, *d* = 0.193, were not significant. The multiple comparison demonstrated the largest contribution of the 90° component compared with 45°, *t*(29) = 9.96, *p* < 0.001, *d* = 1.616, and 180°, *t*(29) = 10.38, *p* < 0.001, *d* = 2.030). The 45° and 180° components were not significantly different from each other, *t*(29) = 1.80, *p* = 0.082, *d* = 0.437.

In summary, the influence of visual periodicity cues on the SVV error increased with room aspect ratio size. We found no evidence that the influence of the room aspect ratio was specific to a particular periodicity. Finally, the contribution of the 90° component was much greater than that of the 45° and 180° components.

### Experiment 2

**Figure 5.**
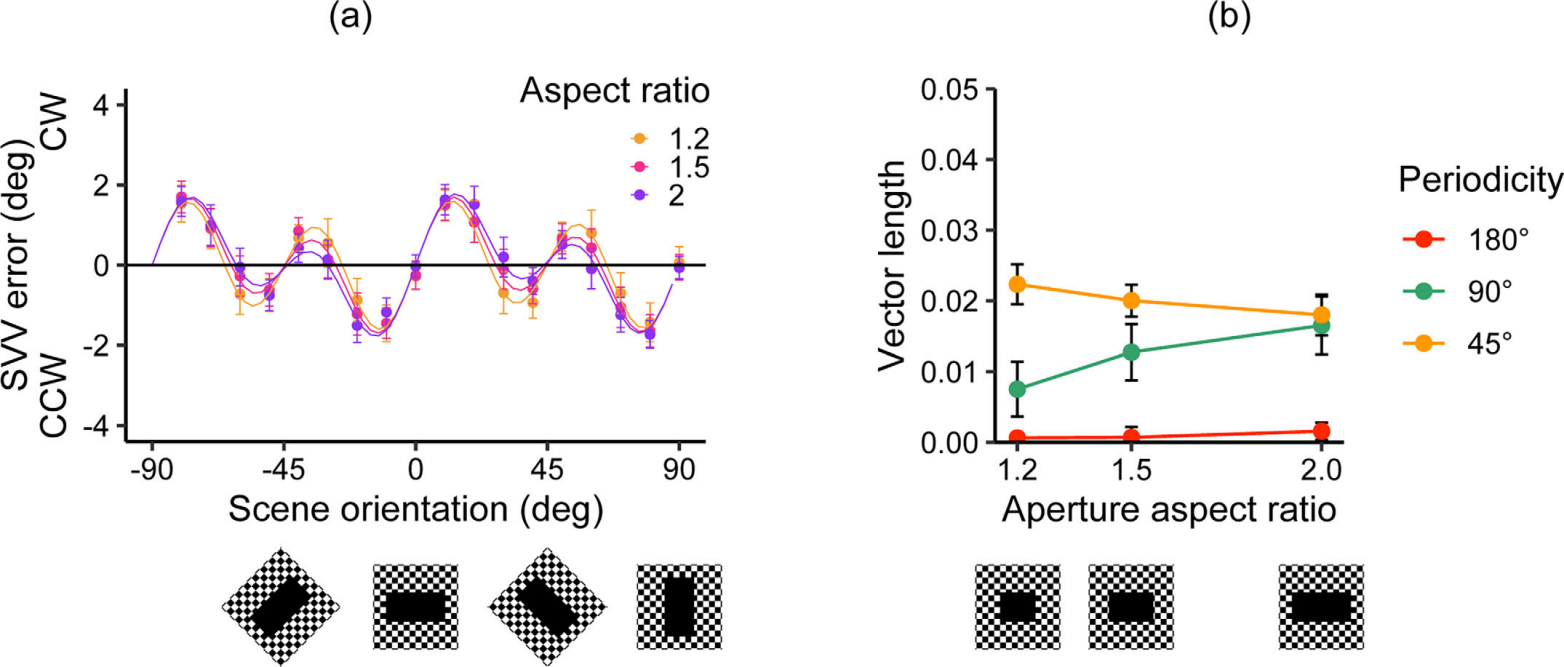
Results of Experiment 2. (**a**) Mean SVV errors as a function of scene orientation. The positive and negative values of the SVV errors indicate that the adjusted rod was tilted CW or CCW, respectively, relative to the gravitational vertical. Data for each aspect ratio were mean centered. The curves indicate the best fit of the model to each aspect ratio. (**b**) Best-fit parameters of the visual vector lengths of the three periodicities for each aperture aspect ratio were obtained. Error bars depict the standard errors of the mean across participants.

Figure 5a illustrates the mean SVV errors with a rectangular aperture stimulus. Figure 5b plots the mean lengths of the visual vectors. The vector lengths of the 180° component were close to zero for all aperture aspect ratios, whereas the lengths of the 45° and 90° components were substantial. The 45° component had the largest vector lengths, which decreased with aspect ratio size.

A two-way repeated-measures ANOVA with periodicity (45°, 90°, and 180°) and aperture aspect ratios (1.2, 1.5, and 2) as within-participant variables revealed no significant main effect of aperture aspect ratio, *F*(1.97, 57.02) = 1.99, *p* = 0.147, η^2^ = 0.002, but a significant main effect of periodicity, *F*(1.51, 43.73) = 13.73, *p* < 0.001, η^2^ = 0.204, and a significant interaction between them, *F*(3.13, 90.79) = 5.78, *p* = 0.001, η^2^ = 0.0167. Subsequent analysis revealed that the 45° component decreased with aperture aspect ratio size, *F*(1.96, 56.93) = 3.32, *p* = 0.044, η^2^ = 0.015, and that the 90° component increased with aperture aspect ratio size, *F*(1.91, 55.28) = 8.29, *p* < 0.001, η^2^ = 0.029, but not for the 180° component, *F*(1.68, 48.76) = 0.20, *p* = 0.780, η^2^ = 0.005. Multiple comparisons of the aperture aspect ratios for the 45° component showed no significant differences: 1.2 versus 1.5, *t*(29) = 1.35, *p* = 0.187, *d* = 0.157; 1.2 versus 2, *t*(29) = 2.45, *p* = 0.062, *d* = 0.277; 1.5 versus 2, *t*(29) = 1.29, *p* = 0.208, *d* = 0.133. Multiple comparisons for the 90° component revealed a smaller vector length for the shortest aperture aspect ratio (1.2) than for the longer aspect ratio conditions: 1.2 versus 1.5, *t*(29) = 2.28, *p* = 0.030, *d* = 0.243; 1.2 versus 2, *t*(29) = 3.79, *p* = 0.002, *d* = 0.410. The difference between the 1.5 and 2 ratios was marginally insignificant, *t*(29) = 1.92, *p* = 0.064, *d* = 0.171. Periodicity showed significant simple main effects for all aspect ratio conditions: 1.2, *F*(1.61, 46.82) = 16.29, *p* < 0.001, η^2^ = 0.266; 1.5, *F*(1.54, 44.6) = 13.41, *p* < 0.001, η^2^ = 0.222; 2, *F*(1.55,44.98) = 8.66, *p* = 0.002, η^2^ = 0.176. Multiple comparisons of periodicity for each aspect ratio demonstrated larger vector lengths for the 45° component than the 90° and 180° components at an aperture aspect ratio of 1.2, whereas the 1.5 and 2 aspect ratios showed that the 180° component had smaller vector lengths than the 45° and 90° components (Supplementary Table S1).

In summary, when presenting frontoparallel surface stimuli we observed an increase in the 90° component corresponding to aperture aspect ratio size, whereas the 180° component was nearly unchanged, remaining close to zero for all aspect ratios. The strongest contribution came from the 45° component at the smallest aspect ratio. The extent of this contribution tended to a decrease with aspect ratio size.

### Experiment 3

**Figure 6.**
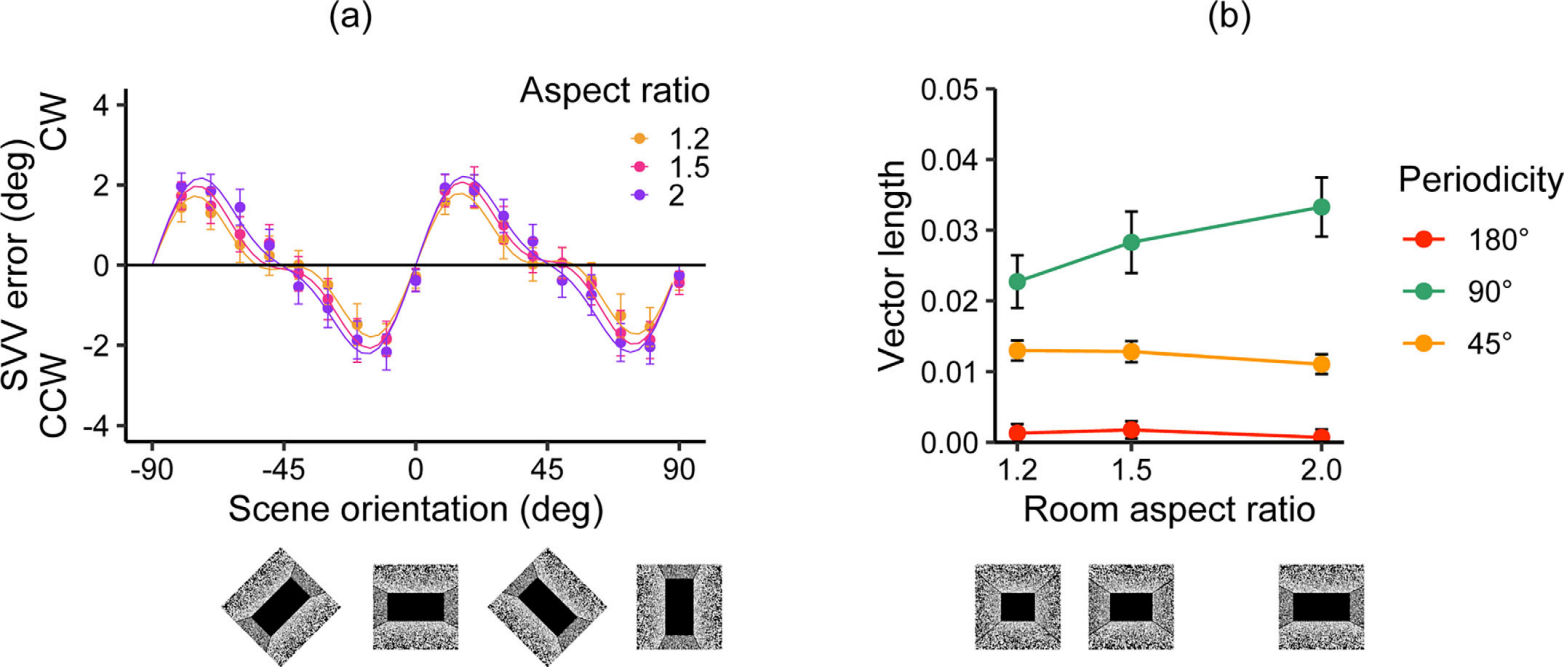
Results of Experiment 3. (**a**) Mean SVV errors as a function of scene orientation. The positive and negative values of the SVV errors indicate that the adjusted rod was tilted CW or CCW, respectively, relative to the gravitational vertical. Data for each aspect ratio were mean centered. The curves indicate the best fit of the model to each data point. (**b**) Best-fit parameters of the visual vector lengths of the three periodicities for each room aspect ratio were obtained. Error bars show the standard errors of the mean across participants.

Figure 6a shows the mean SVV errors for the random dot texture stimulus. Figure 6b plots the mean lengths of the visual vectors. The vector lengths of the 180° component were nearly at zero for all room aspect ratios, whereas the lengths of the 45° and 90° components were substantial.

A within-subject ANOVA, with periodicity (45°, 90°, and 180°) and room aspect ratio (1.2, 1.5, and 2) as variables, revealed significant main effects of aspect ratio, *F*(1.96, 56.86) = 4.65, *p* = 0.014, η^2^ = 0.004, and periodicity, *F*(1.24, 35.91) = 37.94, *p* < 0.001, η^2^ = 0.375, as well as a significant interaction between them, *F*(3.36, 97.41) = 13.50, *p* < 0.001, η^2^ = 0.016. Subsequent analysis revealed aspect ratio to have a significant simple main effect for the 90° component, *F*(1.93,56.07) = 21.84, *p* < 0.001, η^2^ = 0.037, but not for the 45° component, *F*(1.83, 53.1) = 1.56, *p* = 0.220, η^2^ = 0.013, or the 180° component, *F*(1.69, 48.97) = 0.29, *p* = 0.713, η^2^ = 0.004. Multiple comparisons of the aspect ratios for the 90° component revealed that vector lengths increased as the aspect ratio increased: 1.2 versus 1.5, *t*(29) = 3.47, *p* = 0.002, *d* = 0.232; 1.2 versus 2, *t*(29) = 7.22, *p* < 0.001, *d* = 0.464; 1.5 versus 2, *t*(29) = 2.90, *p* = 0.007, *d* = 0.213. In all aspect ratio conditions, periodicity had significant simple main effects: 1.2, *F*(1.32, 38.15) = 25.92, *p* < 0.001, η^2^ = 0.312; 1.5, *F*(1.27, 36.97) = 30.52, *p* < 0.001, η^2^ = 0.351; 2, *F*(1.3, 37.65) = 47.06, *p* < 0.001, η^2^ = 0.479. Multiple comparisons of the periodicity for each aspect ratio demonstrated the largest vector length for the 90° component, followed by the 45° component, with the smallest vector length for the 180° component for all aspect ratios (see Supplementary Table S2).

In summary, we observed a clear increase in the 90° component of the SVV error corresponding to an increase in aspect ratio size. Conversely, the 180° component barely contributed to the SVV error for all aspect ratios. The 45° component made a moderate contribution to the error.

### Comparisons among Experiments 1, 2, and 3

**Figure 7.**
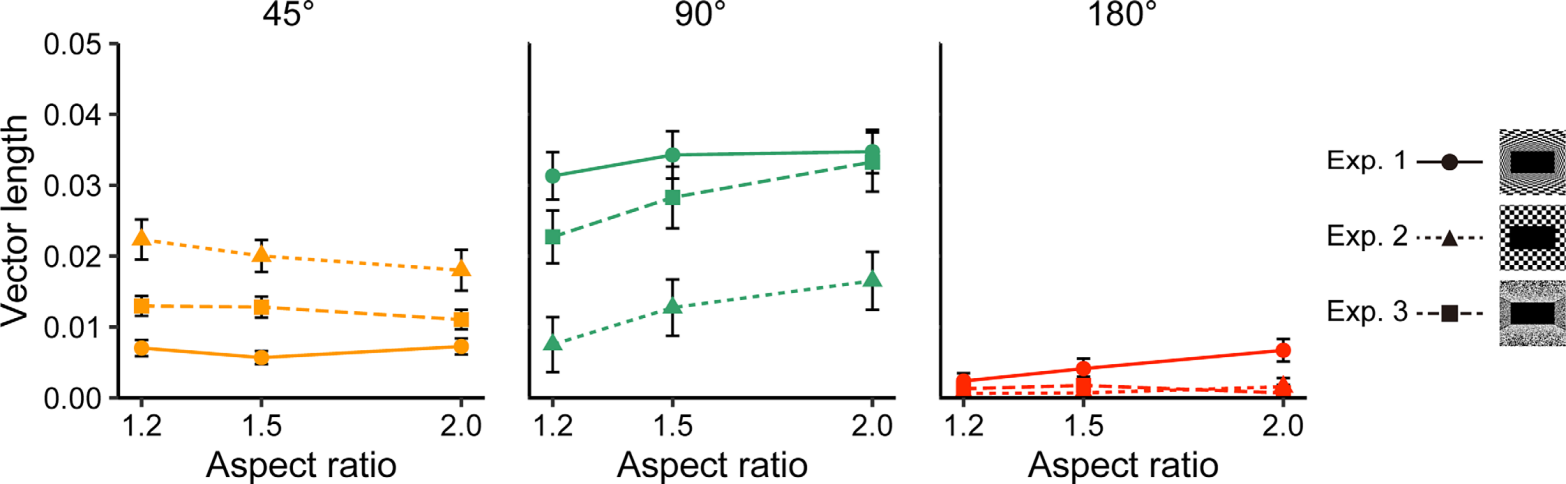
Comparisons of results across Experiments 1, 2, and 3. Each dot represents the mean best-fit parameter for the vector lengths of the three visual periodicities for each aspect ratio and spatial structure from each participant. Error bars represent the standard errors of the mean across participants.

To investigate the influence of spatial structure on the pattern of SVV errors, we combined the vector length data obtained from Experiments 1, 2, and 3 (Figure 7). The 180° component appeared substantial and increased with aspect ratio size in Experiment 1, but in Experiments 2 and 3 it was nearly at zero and did not show a clear increase with aspect ratio size. The 90° component was smaller in Experiment 2 than in Experiments 1 and 3. In contrast, the 45° component in Experiment 2 was the greatest of the three experiments, followed by Experiment 3, and the 45° component was the smallest in Experiment 1.

To obtain statistical support for the effect of spatial structure, we performed a three-way mixed-model ANOVA, with periodicity (45°, 90°, and 180°) and aspect ratio (1.2, 1.5, and 2) as within-participant factors and spatial structure (checkerboard room in Experiment 1, frontoparallel plane in Experiment 2, or random dot texture room in Experiment 3) as a between-participant variable. The results indicated a significant three-way interaction, *F*(5.98, 532.02) = 2.49, *p* = 0.022, η^2^ = 0.003, and significant two-way interactions: spatial structure × periodicity, *F*(2.59, 230.25) = 15.55, *p* < 0.001, η^2^ = 0.113; periodicity × aspect ratio, *F*(3.27, 291.2) = 13.34, *p* < 0.001, η^2^ = 0.009. The other statistical results from our ANOVA analyses and subset analyses for each periodicity are documented in the Supplementary Tables S3 to S6.

## General discussion

### Summary

This study aimed to investigate how the transverse expansion of an indoor scene contributes to the SVV error. We conducted three experiments in a VR environment, each presenting different spatial structures. We used a rectangular checkerboard room (Experiment 1), a frontoparallel plane with a checkerboard background and a rectangular aperture (Experiment 2), and a rectangular random dot texture room (Experiment 3). The width-to-height aspect ratio was manipulated, with the expectation that this would modulate the periodic components of the SVV error across scene orientations.

In Experiment 1, the periodic components of the SVV error increased with aspect ratio size, but there was no significant interaction between periodicity and aspect ratio. In Experiment 2, where depth information was removed, only the 90° component increased with aspect ratio size, whereas the 180° component almost disappeared. The 45° component made the largest contribution at smaller aspect ratios and this contribution decreased with aspect ratio size. In Experiment 3, the 90° component increased with aspect ratio size, whereas the 180° component remained close to zero.

Comparing the results of the three experiments highlighted the role of depth cues. In Experiment 1, where depth cues were the richest, with an explicit linear perspective, the 180° component was larger than in Experiments 2 and 3. The 90° component was larger in Experiments 1 and 3 than in Experiment 2, which had the poorest depth cues without the transverse planes. In contrast, the 45° component was larger in Experiment 2 than in Experiments 1 and 3.

### Relevance of depth cues to the 180° component

The spatial structure in Experiment 2 excluded the transverse surface, and the linear perspective of the transverse surface was eliminated in Experiment 3. Thus, the increased contribution of the 180° component with aspect ratio size in Experiment 1 compared with Experiments 2 and 3 illustrates the relevance of the extended transverse surface associated with horizontal cues (Fujimoto & Ashida, 2022; Haji-Khamneh & Harris, 2009; Harris et al., 2011). The role of the transverse surface found here is consistent with previous research that only reported the 90° component with a 2D line inducer (Li & Matin, 2005a; Vingerhoets et al., 2009). It may be that extending the 2D line components alone might be not sufficient to enhance the 180° component.

The random dot texture room used for Experiment 3 also drastically reduced the 180° component, highlighting the importance of a linear perspective. Many studies have demonstrated the importance of a linear perspective when interpreting a spatial structure, as demonstrated in an Ames room (Day, 1993; Gregory, 1987; Ittelson, 1952) and from a reverse perspective (Papathomas & Hughes, 2019; Wade & Hughes, 1999; Yoo, Lee, & Joo, 2023). A linear perspective itself could be used to locate the true horizon if multiple vanishing points are aligned. In Experiment 1, one could recognize implicit vanishing points made by the diagonals of the checkerboard squares. Although diagonals on the floor, ceiling, and walls make vanishing points aligned either horizontally or vertically, increased area of the floor and ceiling with larger aspect ratios might bias the horizontal alignment, providing a cue to the horizon.

The reduction of the 180° component may be partially attributed to the greater compressibility of the perceived depth of the scene in Experiments 2 and 3 compared to that in Experiment 1. In Experiments 2 and 3, there was a greater likelihood of distance underestimation than in Experiment 1, which had stronger depth cues. The reduction of the perceived surface area might have led to a reduction in the effect of the transverse surface on the 180° component. This might also have decreased the apparent size of the spatial structure. However, this is unlikely to have caused the reduced SVV error, as previous studies suggest that retinal size is more crucial than observation distance or the apparent size of the frame in the rod-and-frame illusion (Ebenholtz, 1977; Ebenholtz & Callan, 1980).

### Role of ecological relevance in the 180° component

The increased contribution of the 180° component in Experiment 1 can be interpreted from an ecological perspective. Terrestrial animals rely heavily on the ground surface for spatial orientation (Gibson, 1950; Gibson, 1979). The ground can be defined as a reliable support surface that is horizontal (perpendicular to gravity), flat, sufficiently extended, and rigid (Gibson, 1979). Harris, Jenkin, Jenkin, Dyde, and Oman (2010) found that a large surface in an indoor scene is frequently selected as a support surface, demonstrating the role of surface expansion in ground recognition. Assuming that the 180° component corresponds to the horizontal cue (Fujimoto & Ashida, 2022; Haji-Khamneh & Harris, 2009; Harris et al., 2011), our result suggests that the relative relevance of a surface as a support increases with the aspect ratio, providing an implicit horizontal cue and leading to the increased contribution of the 180° component. The specific relationship between the intensity of surface relevance and the SVV error remains to be determined in future research.

### Possible confounders of aspect ratio

The results of Experiment 1 showed no evidence that the effects of room aspect ratio are specific to the 180° component, but this may be due to confounding manipulations of the visual stimuli. The increase in the 90° component in the current study might be attributable to the increase in length of the line components with aspect ratio size (Alberts et al., 2016; Cian, Esquivié, Barraud, & Raphel, 1995; Ebenholtz, 1977; Ebenholtz & Benzschawel, 1977; Ebenholtz & Callan, 1980; Ebenholtz & Glaser, 1982; Li & Matin, 2005a; Li & Matin, 2005b). Although this could explain the increase in the 180° component in Experiment 1 as well, it is unlikely for two reasons. First, the 180° component did not increase when the depth cues were reduced in Experiments 2 and 3, despite the clear increase in the 90° component. Second, previous studies have only reported the 90° component with 2D line inducers, even when the inducer length was extended to peripheral vision (Li & Matin, 2005a; Vingerhoets et al., 2009). These points suggest that inducing the 180° component requires additional cues to an extensive transverse surface. Thus, increasing the aspect ratio of the room could have simultaneously affected the 90° and 180° components, confounding the influence of the aspect ratio on the two components in Experiment 1.

### Effects of spatial structure on the 90° component

The 90° component was larger when room-structured scenes were presented in Experiments 1 and 3, demonstrating the importance of spatial depth structure to this component. This result might have been caused by surface tilt information, which was absent in Experiment 2. In addition, adding depth cues might facilitate immersion and presence in the scene, enhancing the weight of visual orientation cues, although the specific spatial structures (e.g., openness, navigability, roughness) (Oliva, 2005; Oliva & Torralba, 2001; Oliva & Torralba, 2006) that enhance immersion remain unknown.

### Involvement of the virtual axis of symmetry with the 45° component

We found a substantial contribution to the SVV error by the 45° component, although previous studies using the weighted vector model have not considered this periodicity (Alberts et al., 2016; Alberts et al., 2018; Fujimoto & Ashida, 2022; Haji-Khamneh & Harris, 2009; Harris et al., 2007; Harris et al., 2011; Medendorp, Alberts, Verhagen, Koppen, & Selen, 2018; Mittelstaedt, 1986; Vingerhoets et al., 2009). Although portions of the 45° component could be artifacts of the harmonic distortion of the 180° and 90° components, its amplitude should have been much smaller than that of the latter two components in Experiment 2. The contribution of this component might also be explained by the influence of the virtual axes of symmetry of the scene (Beh & Wenderoth, 1972; Beh et al., 1971; Ebenholtz & Benzschawel, 1977; Wenderoth, 1973; Wenderoth, 1978; Wenderoth & Beh, 1977; Wenderoth & Johnstone, 1987; Wenderoth, Johnstone, & Van der Zwan, 1989; Wenderoth & Van Der Zwan, 1991). Beh et al. (1971) reported an inverse rod-and-frame illusion direction when the frame was oriented around 45°. A series of studies by Wenderoth et al. found that diagonals of the frame can be recognized as virtual axes of symmetry. This further influences the angular function of the SVV error besides the explicit line components (Daini, Wenderoth, & Smith, 2003; Wenderoth, 1973; Wenderoth & Johnstone, 1987; Wenderoth et al., 1989; Wenderoth & Van Der Zwan, 1991). Simulation of real and virtual axis effects can inevitably expect higher order component effects (see figure 6 in Wenderoth & Van Der Zwan, 1991; see also the right half of our Figure 5a). Although the diagonals of the rectangular frame are not technically axes of symmetry, some of the 45° component could have originated from the influence of the virtual axes of the rectangular room. This study provides some empirical evidence of the virtual axis effect by revealing the contribution of the 45° component of the SVV error.

We found that the 45° component tended to decrease with aspect ratio size, especially in Experiment 2. A larger aperture aspect ratio should distort diagonals further out of symmetry, possibly reducing the influence of the virtual axes of symmetry. It is possible that the distortion of diagonal symmetry by large aspect ratios added subharmonic noise to the other periodicities. The 180° component appeared to be immune to the virtual axis effect, as the 180° component did not correlate with the 45° component.

Whereas Experiments 1 and 3 showed that the largest contribution to the SVV error came from the 90° component, the 45° component made the strongest contribution in Experiment 2, especially with the smallest aspect ratio. It may be that observer immersion in the room inhibits recognition of the diagonal axes of the room in Experiments 1 and 3.

### Limitations of the study

The disparity cues presented via our virtual reality HMD may have been inaccurate for some participants due to the presentation of visual stimuli using a fixed interpupillary distance. Although disparity cues are commonly examined over short distances (Cormack, 1984; Ono & Comerford, 1977), several studies suggest that they can also enhance distance estimation in extrapersonal spaces (Allison, Gillam, & Vecellio, 2009; Cormack, 1984; Palmisano, Gillam, Govan, Allison, & Harris, 2010). In the context of the perceived spatial properties of a ground surface, Allison, Gillam, and Palmisano (2009) demonstrated the superiority of stereoscopic over monocular judgments of surface slant. This further implies that vertical perception of the pitch axis largely depends on binocular cues. Given our finding that perceived surface expansion may contribute to vertical perception, disparity cues could also enhance the estimation of surface depth. Future research should investigate the effects of disparity cues by presenting scenes with and without binocular disparity.

The 180° component of the SVV error was much smaller, even in Experiment 1, than that found in our previous study (Fujimoto & Ashida, 2022), in which we presented an indoor scene using identical apparatus and an aspect ratio of 2:1. The estimated vector length for the 180° component in Experiment 1 decreased to approximately 26% relative to our previous study, whereas the estimated vector length for the 90° component moderately decreased to approximately 70%. These results suggest that factors other than surface area are crucial for the 180° component. In our previous study, we presented a furnished room with wood flooring and vinyl wallpaper to distinguish the floor surface from the other surfaces in the room. The differences between the results of our previous study and the current study suggest that the naturalness of the scene might also affect the SVV error.

## Conclusions

This study suggests that the depth and width of a scene play crucial roles in the periodic components of the SVV error. First, the increased 180° component with increased room aspect ratio in Experiment 1, in which rich depth cues were provided, suggests the ecological relevance of surface expansion in the transverse direction. This is associated with horizontal perception. Second, the 90° component increased with aspect ratio size, and room-structured scenes enhanced this component, suggesting the importance of the depth and width of the scene to the component. Finally, we revealed a substantial contribution to the SVV error by the 45° component, possibly originating from the influence of virtual symmetry axes within the scene. As with the other components, the 45° component was modulated by the aspect ratio and spatial structure. Together, these findings highlight the importance of the transverse expansion of a scene for verticality perception.

## Supplementary Material

Supplement 1
