# Supplementary material for: Influence of scene aspect ratio and depth cues on verticality perception bias
Source: J Vis. 2024 Jul 19;24(7):12. doi: 10.1167/jov.24.7.12 (PMC11282478; doi:10.1167/jov.24.7.12)
Supplement: Supplement 1 [file jovi-24-7-12_s001.docx]

Supplementary Material


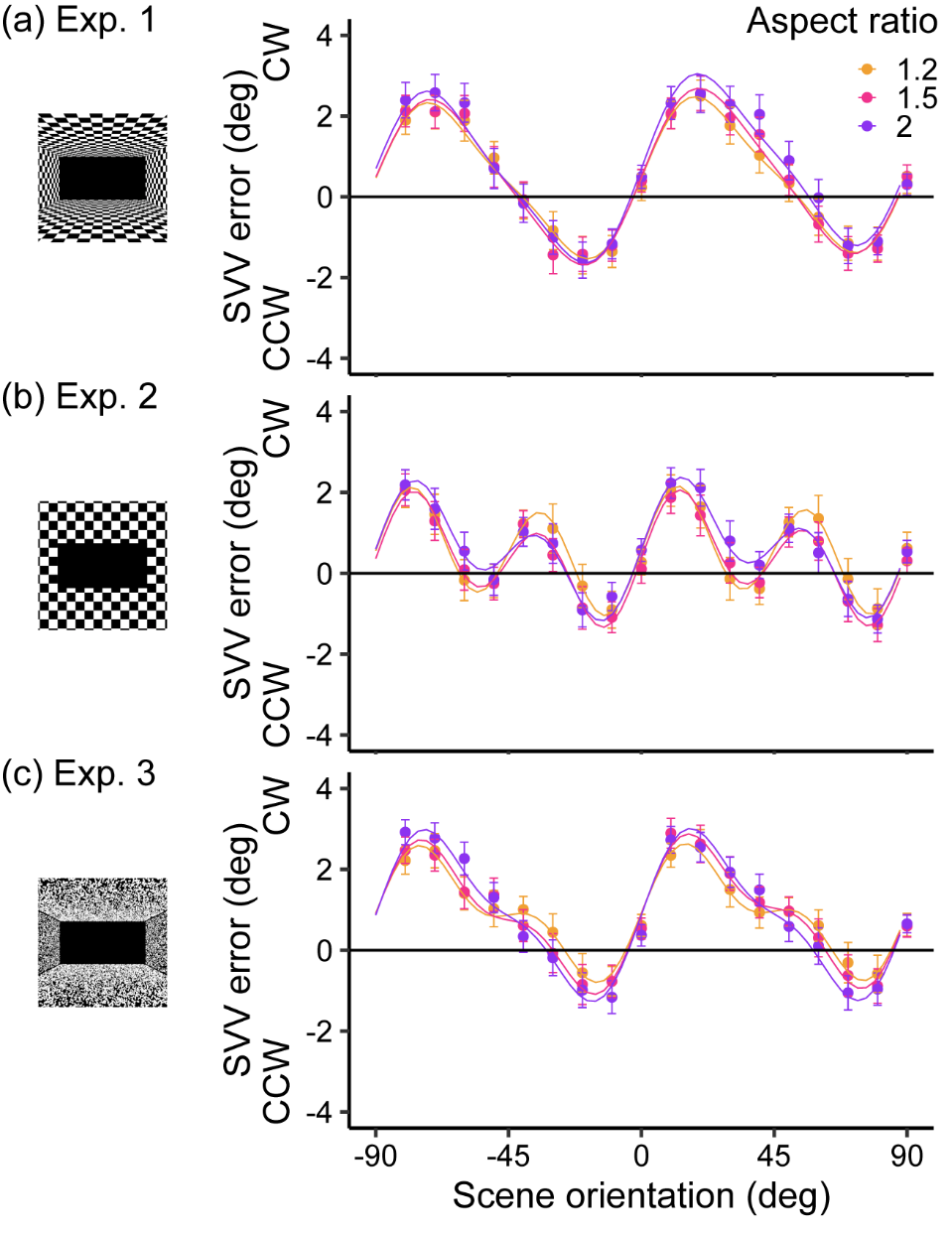


Figure S1. Mean SVV errors as a function of scene orientation without mean-centering. Positive and negative SVV error values correspond to CW or CCW rod adjustments, respectively, relative to the gravitational vertical. Data were not mean-centered. The curves demonstrate the best fit of the model to the data.

Table S1. Multiple comparison of periodicity for each aspect ratio in Experiment 2.

| Aspect Ratio | Pair | *df* | *t* | Adjusted *p* | *d* |  |  |
| --- | --- | --- | --- | --- | --- | --- | --- |
| 1.2 | 45° vs. 90° | 29 | 3.22 | .003 | 0.795 | ** | 90° < 45° |
|  | 45° vs. 180° | 29 | 7.46 | .000 | 1.948 | *** | 180° < 45° |
|  | 90° vs. 180° | 29 | 1.74 | .092 | 0.461 |  |  |
|  |  |  |  |  |  |  |  |
| 1.5 | 45° vs. 90° | 29 | 1.67 | .107 | 0.409 |  |  |
|  | 45° vs. 180° | 29 | 7.57 | .000 | 1.836 | *** | 180° < 45° |
|  | 90° vs. 180° | 29 | 2.93 | .007 | 0.721 | **** | 180° < 90° |
|  |  |  |  |  |  |  |  |
| 2 | 45° vs. 90° | 29 | 0.28 | .781 | 0.077 |  |  |
|  | 45° vs. 180° | 29 | 5.14 | .000 | 1.365 | *** | 180° < 45° |
|  | 90° vs. 180° | 29 | 3.42 | .002 | 0.915 | **** | 180° < 90° |

** *p* < .01, *** *p* < .001

Table S2. Multiple comparison of periodicity for each aspect ratio in Experiment 3.

| Aspect Ratio | Pair | *df* | *t* | Adjusted *p* | *d* |  |  |
| --- | --- | --- | --- | --- | --- | --- | --- |
| 1.2 | 45° vs. 90° | 29 | 3.21 | .003 | 0.499 | ** | 45° < 90° |
|  | 45° vs. 180° | 29 | 6.55 | .000 | 1.590 | *** | 180° < 45° |
|  | 90° vs. 180° | 29 | 5.67 | .000 | 1.365 | ***** | 180° < 90° |
|  |  |  |  |  |  |  |  |
| 1.5 | 45° vs. 90° | 29 | 4.12 | .003 | 0.717 | ***** | 45° < 90° |
|  | 45° vs. 180° | 29 | 6.27 | .000 | 1.481 | *** | 180° < 45° |
|  | 90° vs. 180° | 29 | 6.30 | .000 | 1.405 | ***** | 180° < 90° |
|  |  |  |  |  |  |  |  |
| 2 | 45° vs. 90° | 29 | 5.55 | .000 | 1.204 | ***** | 45° < 90° |
|  | 45° vs. 180° | 29 | 5.86 | .000 | 1.484 | *** | 180° < 45° |
|  | 90° vs. 180° | 29 | 8.10 | .000 | 1.779 | ***** | 180° < 90° |

** *p* < .01, *** *p* < .001

Table S3. Results of a three-way ANOVA with spatial structure as a between-subject factor and periodicity and aspect ratio as within-subject factors.

| Effect | *df* | *F* | *p* | *η2* |  |
| --- | --- | --- | --- | --- | --- |
| Spatial Structure | 1.91,170.19 | 1.41 | .247 | 0.008 |  |
| Periodicity | 1.42,123.79 | 92.63 | .000 | 0.266 | *** |
| Aspect Ratio | 1.89,168.34 | 10.76 | .000 | 0.003 | *** |
| Spatial Structure x Periodicity | 2.59,230.35 | 15.55 | .000 | 0.113 | *** |
| Spatial Structure x Aspect Ratio | 3.14,279.35 | 0.28 | .850 | 0.000 |  |
| Periodicity x Aspect Ratio | 3.27,291.2 | 13.34 | .000 | 0.009 | *** |
| Spatial Structure x Periodicity x Aspect Ratio | 5.98,532.02 | 2.49 | .022 | 0.003 | ** |

** *p* < .01, *** *p* < .001

Table S4. Effects of aspect ratio and spatial structure on the 45° component

| Effect | *df* | *F* or *t* | *p* | *η2* or *d* |  |  |
| --- | --- | --- | --- | --- | --- | --- |
| Spatial Structure | 2,87 | 16.50 | .000 | 0.231 | *** |  |
| Aspect Ratio | 1.96,170.11 | 3.07 | .050 | 0.005 |  |  |
| Spatial Structure x Aspect Ratio | 3.91,170.11 | 1.72 | .150 | 0.006 |  |  |
| Multiple comparison: Exp. 1 vs. 2 | 87 | 5.72 | .000 | 1.319 | *** | Exp. 1 < 2 |
| Multiple comparison: Exp. 1 vs. 3 | 87 | 2.39 | .019 | 1.007 | * | Exp. 1 < 3 |
| Multiple comparison: Exp. 2 vs. 3 | 87 | 3.33 | .001 | 0.733 | ** | Exp. 2 > 3 |

* *p* < .05, ** *p* < .01, *** *p* < .001

Table S5. Effects of aspect ratio and spatial structure on the 90° component

| Effect | *df* | *F* or *t* | *p* | *η2* or *d* |  |  |
| --- | --- | --- | --- | --- | --- | --- |
| Spatial Structure | 2,87 | 9.18 | .000 | 0.159 | *** |  |
| Aspect Ratio | 1.95,169.67 | 25.29 | .000 | 0.020 | *** |  |
| Spatial Structure x Aspect Ratio | 3.9,169.67 | 2.04 | .093 | 0.003 |  |  |
| Multiple comparison: Exp. 1 vs. 2 | 87 | 4.12 | .000 | 1.12 | *** | Exp. 1 > 2 |
| Multiple comparison: Exp. 1 vs. 3 | 87 | 1.04 | .301 | 0.275 |  |  |
| Multiple comparison: Exp. 2 vs. 3 | 87 | 3.08 | .003 | 0.744 | ** | Exp. 2 < 3 |

** *p* < .01, *** *p* < .001

Table S6. Effects of aspect ratio and spatial structure on the 180° component

| Effect | *df* | *F* or *t* | *p* | *η2* or *d* |  |  |
| --- | --- | --- | --- | --- | --- | --- |
| Spatial Structure | 2,87 | 4.47 | .014 | 0.049 | * |  |
| Aspect Ratio | 1.98,172.68 | 1.58 | .208 | 0.008 |  |  |
| Spatial Structure x Aspect Ratio | 3.97,172.68 | 1.51 | .203 | 0.016 |  |  |
| Multiple comparison: Exp. 1 vs. 2 | 87 | 2.69 | .026 | 0.704 | * | Exp. 1 > 2 |
| Multiple comparison: Exp. 1 vs. 3 | 87 | 2.47 | .026 | 0.575 | * | Exp. 1 > 3 |
| Multiple comparison: Exp. 2 vs. 3 | 87 | 0.22 | .826 | 0.064 |  |  |

* *p* < .05
